# Vertical motion history of the island of O‘ahu, Hawaiian Islands, during the last two million years

**DOI:** 10.1038/s41598-025-10350-1

**Published:** 2025-07-21

**Authors:** Michael Toomey, Michael Sandstrom, Kimberly Huppert, Frederick Taylor, Thomas M. Cronin

**Affiliations:** 1 United States Geological Survey, Reston, VA 20192 USA; 2 Department of Geological Sciences, Jackson School of Geosciences, University of Texas at Austin, Austin, TX 78712 USA; 3 University of North Carolina, Chapel Hill, NC 27599 USA; 4 Department of Earth and Atmospheric Sciences, CUNY City College of New York, New York, NY 10031 USA; 5 Institute for Geophysics, Jackson School of Geosciences, University of Texas at Austin, Austin, TX 78758 USA

**Keywords:** Sedimentology, Stratigraphy, Geodynamics

## Abstract

Intraplate hotspots deform the Earth’s lithosphere and shape the morphology of reef-bound islands. This paper constrains the depositional history of the coastal plain of O‘ahu, Hawaii over the past 2 million years using: (1) the strontium isotope stratigraphy of shallow water carbonates (e.g., corals, mollusks) sampled from a 337 m-long drill core; (2) model-predicted vertical motion of the Ewa Coastal Plain that incorporates displacements due to the flexural isostatic response of the lithosphere to loading of each volcano along the Hawaiian Chain as well as O‘ahu’s migration over the Hawaiian Swell. The results of this study indicate that O‘ahu subsided rapidly (~ 0.5 mm/yr) during the early Pleistocene via vertical displacements that our model largely attributes to crustal loading during construction of the Maui Nui complex. An abrupt slowing of subsidence during the past million years was likely caused by the relative progression of volcanism eastward to the island of Hawai‘i and O‘ahu’s migration over the crest of the swell. The morphologic transition from drown reef terraces offshore O‘ahu, to initiation of stable carbonate accumulation at the Ewa Coastal Plain, to uplifted carbonate shorelines at O‘ahu during the Pleistocene has been driven by progressive changes in vertical motion as the island has migrated away from the Hawaiian hotspot.

## Introduction

**Fig. 1:**
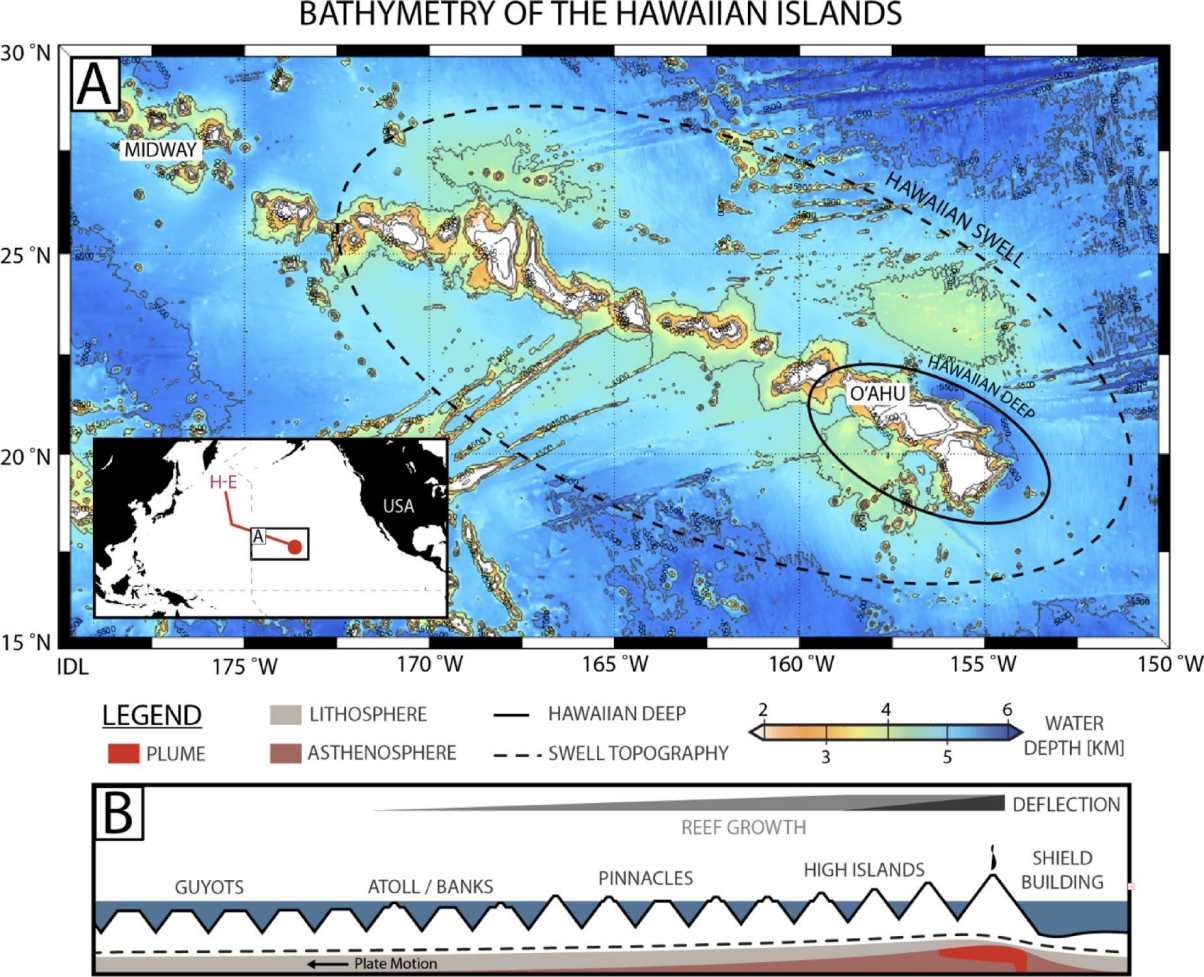
Bathymetry of the Hawaiian Islands hotspot chain. (**A**) Bathymetry of the Hawaiian Islands outlining the Hawaiian ‘Deep’ and ‘Swell.’ Bathymetric data was acquired from the Global Multi-Resolution Topography Data Synthesis^8^. Map was created using the m_map^9^ (https://www-old.eoas.ubc.ca/~rich/map.html) software package (v 1.4) for Matlab. (**B**) Schematic model of the along-chain profile of the Hawaiian Islands highlighting changes in island morphology and their drivers. Note: schematic components are not drawn to scale.

The Hawaiian-Emperor hotspot chain forms a ~ 6000 km-long string of progressively older islands in the central North Pacific Ocean moving northwest from the island of Hawai‘i (20 °N, 155 °W), actively erupting today, to Meiji (53°N, 165°E), which is a deeply submerged (> 3 km below sea level) seamount near the Aleutian Trench (53 °N, 165 °E) that was formed ~ 85 million years ago^1,2^. The morphologic progression in the Hawaiian Islands (Fig. 1A,B) from reef rimed high volcanic islands to pinnacles, carbonate banks and guyots (drowned atolls) is distinct from classical models of atoll development^3^. The imprint of reef drowning is extensive in the Hawaiian Islands (e.g., terraces, guyots) and results from the combined effects of high amplitude sea-level oscillations with: (1) marginal conditions for coral growth^4^, (2) wave erosion from high-latitude storms^5^ and (3) rapid rates of subsidence during the early phases of volcanic island emplacement^6,7^.

During volcanic shield building of oceanic islands, nearby islands and the adjacent sea floor subside as the lithosphere is depressed by the load of the growing volcano^6^. For example, this can be seen through the warping of drowned reef terraces offshore the island of Maui (Hawaii) which formed near sea level but now dip toward the actively growing island of Hawai‘i and inward toward the axis of the Hawaiian Ridge^10^. However, older islands that have been transported by plate motion further to the northwest, and onto the flexural bulge generated by the loading of younger Hawaiian volcanoes, experience uplift^11,12^. In addition to vertical motion from crustal loading, islands along intraplate hotspots may experience uplift or subsidence due to their migration with plate motion (e.g., Fig. 1A,B) over the broad bathymetric high (swell) that surround oceanic hotspots^13–15^. The bathymetry of the seafloor near hotspot swells rises from dynamic uplift of the lithosphere by ascending plume material^16^, and decreases hundreds to more than a 1000 km (km) downstream. Vertical tectonic displacements of islands along such intraplate hotspot chains also result from various other processes including intrusive volcanism and isostatic adjustment following flank collapse^17^.

**Fig. 2:**
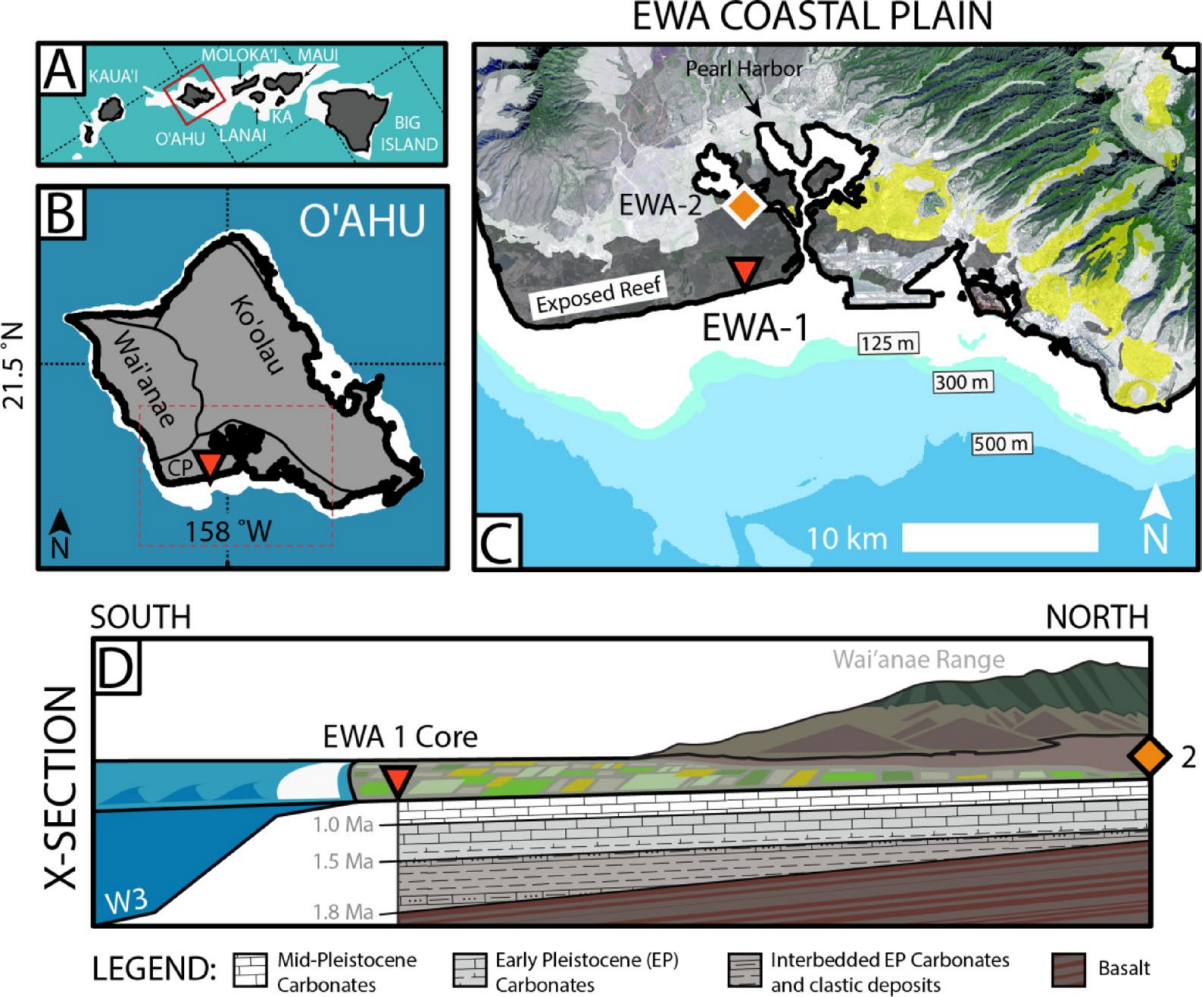
Location and stratigraphy of the O‘ahu Coastal Plain. (**a**) Map of the Hawaiian Islands. Red box outlines extent of panel b. Kaho‘olawe shown by abbreviation ‘KA.’ (**b**) Geography of O‘ahu showing the approximate boundaries of Wai‘anae, Ko‘olau and Coastal Plain (CP) deposits based on U.S. Geological Survey mapping^21^. Map in panels A, B and C were made using ArcGIS 10 (www.arcgis.com). Red dashed box outlines extent of panel c. (**c**) Core site location is given by red star. Yellow and grey shading shows extent of Honolulu volcanics and alluvial deposits, respectively. Aerial imagery acquired from NASA. Surficial geology mapped by U.S. Geological Survey^21^. (**d**) Generalized schematic cross-section of the Ewa Coastal Plain, looking west at Waianae Range, on the basis of new ^87^Sr/^86^Sr ages from EWA-1 and the biostratigraphy of Resig.

The island of O‘ahu (Hawaiian Islands) is thought to be experiencing uplift on the flexural bulge^18^ generated by construction of the island of Hawai‘i while being transported away from the crest of the Hawaiian Swell with plate motion (Figs. 1, 2). Rapid subsidence of O‘ahu during construction of the Maui-Nui complex (Moloka‘i, Lāna‘i, Kaho‘olawe and Maui, Fig. 2A), which is thought to have occurred ~ 1–2 million years ago^19^, is recorded by relict reef shorelines. This paper presents ^87^Sr/^86^Sr measurements sampled downcore along a 337-m (m) long borehole drilled through the Ewa Coastal Plain of the island of O‘ahu^20^ (Fig. 2B–D) in order to reconstruct the vertical motion history of the island during the past 2 million years (Myrs). This new record is ~ 1.5 million years longer than is known from uplifted shorelines elsewhere on the island and is among the oldest vertical motion records in the Hawaiian Islands, providing a unique opportunity to examine the migration of O‘ahu from the flexural moat of the Maui-Nui loading onto the forebulge of the island of Hawai‘i.

## Study site

O‘ahu is a highly eroded double-coned island ~ 400 km NW of the youngest island in the chain, Hawai‘i (Fig. 2B,C), and was built between ~ 3.9 and 1.8 Ma^19^. The Ewa Coastal Plain forms a wedge of clastic and reefal sediments (~ 2–8 km wide) between the Wai‘anae and Ko‘olau volcanic ranges in southern O‘ahu (Fig. 2B). The Ewa Coastal Plain continues as a low angle (~ 1% grade) submarine ramp for ~ 1–5 km offshore, eventually terminating at ~ 20–40 m below sea level (mbsl). Shallow, submerged middle-to-late Pleistocene paleo-shorelines have previously been identified around O‘ahu^22^, including at least nine drowned reef terraces, submerged up to ~ 1 km^10^, thought to have formed as the result of reef drowning during glacial terminations^23^. Continuing offshore along a transect perpendicular to the Hawaiian Chain, the seafloor plunges ~ 4.7 km into the Hawaiian Deep before arching up ~ 500 m (~ 250 km off axis). Equivalent age (~ 80–100 Ma^24^) ocean crust beyond the swell (~ 600 km off axis) is > 1 km deeper than on the Hawaiian Swell near O‘ahu (e.g., Fig. 1A).

Stearns and Chamberlain^20^ collected two continuous cores (> 85% recovery^25^) through the Ewa Coastal Plain during the spring of 1965 CE (NSF GP3545). These cores (Ewa No. 1, Ewa No. 2) are now archived at the University of Hawai‘i Marine Center in Honolulu. This study focuses on Ewa No. 1 (referred to herein as EWA-1), a 337-m (1107-ft.) long core drilled ~ 0.2 km inland from Ewa Beach at approximately 21.3 °N, 158.0 °W, and ~ 2 m above sea level (masl). Ewa No. 2 (21.4 °N, 158.0 °W) was collected ~ 3 km further inland (North) along the 158th parallel adjacent to Pearl Harbor (Fig. 2C,D). A stratigraphic transect from EWA-1 to 2, inferred by Resig^25^, shows a series of inter-bedded, shallow-water, limestones, mudstones and coarse siliciclastic sediments (many of which taper shoreward), that overlie basaltic basement. Many of the biofacies identified by Resig^25^ are indicative of shallow-water lagoonal environments and are comparable to assemblages found in ~ 10–15 m of water offshore O‘ahu today. Alternations between lithologies and faunal assemblages (foraminifera, ostracodes, bryozoans, mollusks, barnacles) define at least eight distinct transgressional sequences in EWA-1^25^.

## Methods

**Fig. 3:**
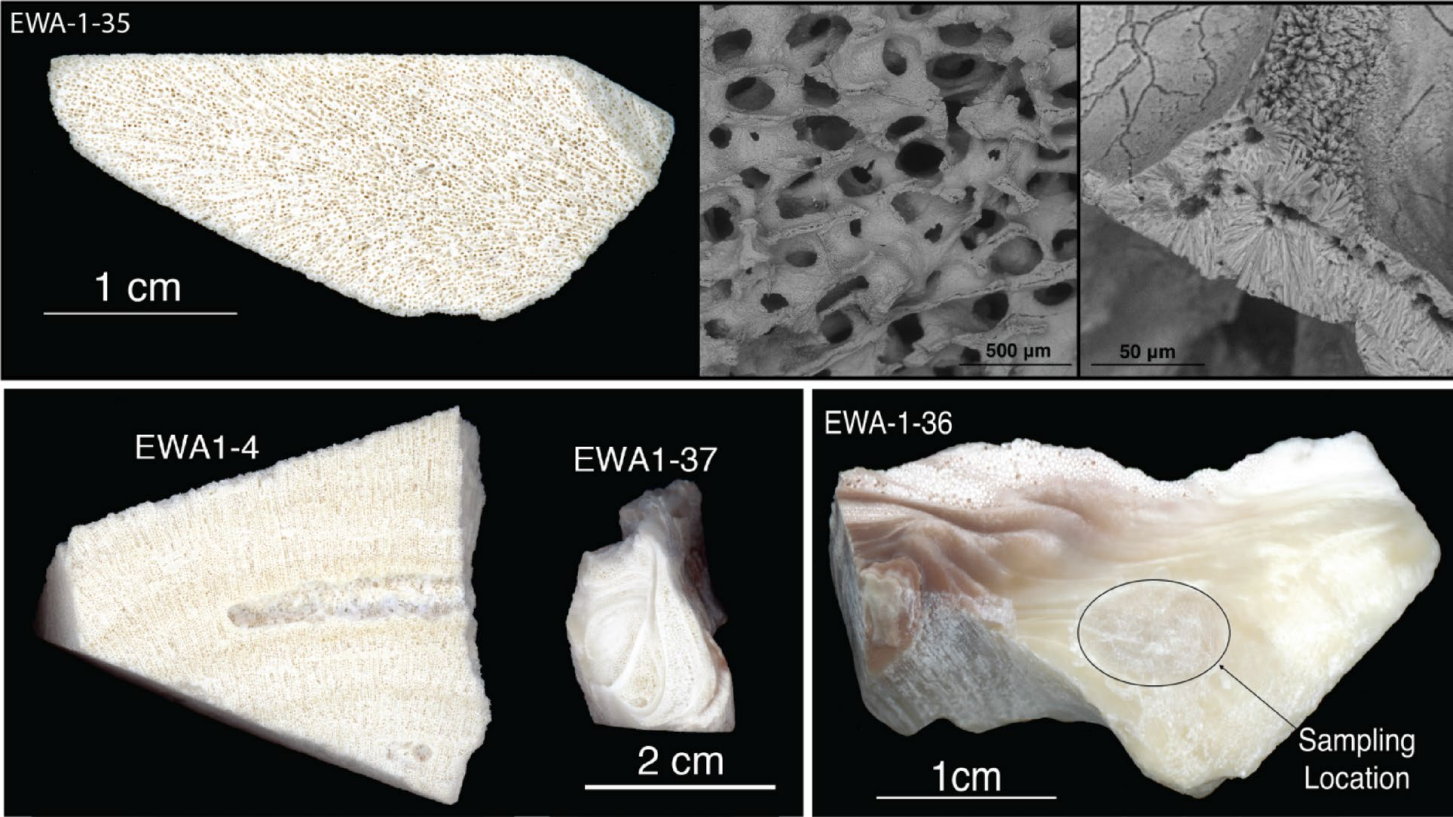
(Top) Coral slab and SEM images (144 × and 1400x) of sample EWA-1-35. (Bottom) From left to right, cross-section images of samples EWA-1-4, 37 and 36.

In order to improve constraints on the depositional history of the Ewa Coastal Plain, corals, mollusks, coralline algae, and biogenic sediment were extracted from EWA-1 during June 2015 and prepared for ^87^Sr/^86^Sr stratigraphy dating. Each sample (e.g., Fig. 3) was: (1) trimmed using a wet saw and (2) ultrasonically cleaned in distilled water and then (3) 125 to 200 mg of powder was extracted using a handheld drill. Possible diagenetic alteration of coral samples was additionally assessed using X-ray diffraction (Table 1). Strontium isotope analyses were performed using conventional techniques on a Thermal Ionization Mass Spectrometer (TIMS) at the Jackson School of Geosciences Isotope Clean Lab, at the University of Texas at Austin (NBS987 = 0.710240 ± 0.000013, n = 19). Two sample analyses (Table 1) were replicated at SUNY Stony Brook (NBS987 = 0.710242 ± 0.000011, n = 21, for sample EWA1-25, and 0.710243 ± 0.000007, n = 6, for EWA1-35). For sample EWA 1–37 (Table 1), the direction and possible magnitude of ^87^Sr/^86^Sr alteration were tested on the basis of measurements from the initial ammonium acetate wash compared to that following acetic acid dissolution of inner carbonate material^26^. All ^87^Sr/^86^Sr ratios were converted to calendar years using the strontium isotope stratigraphy (SIS) seawater calibration curve originally presented by McArthur et al.^27^, updated version 5.

**Table 1:**
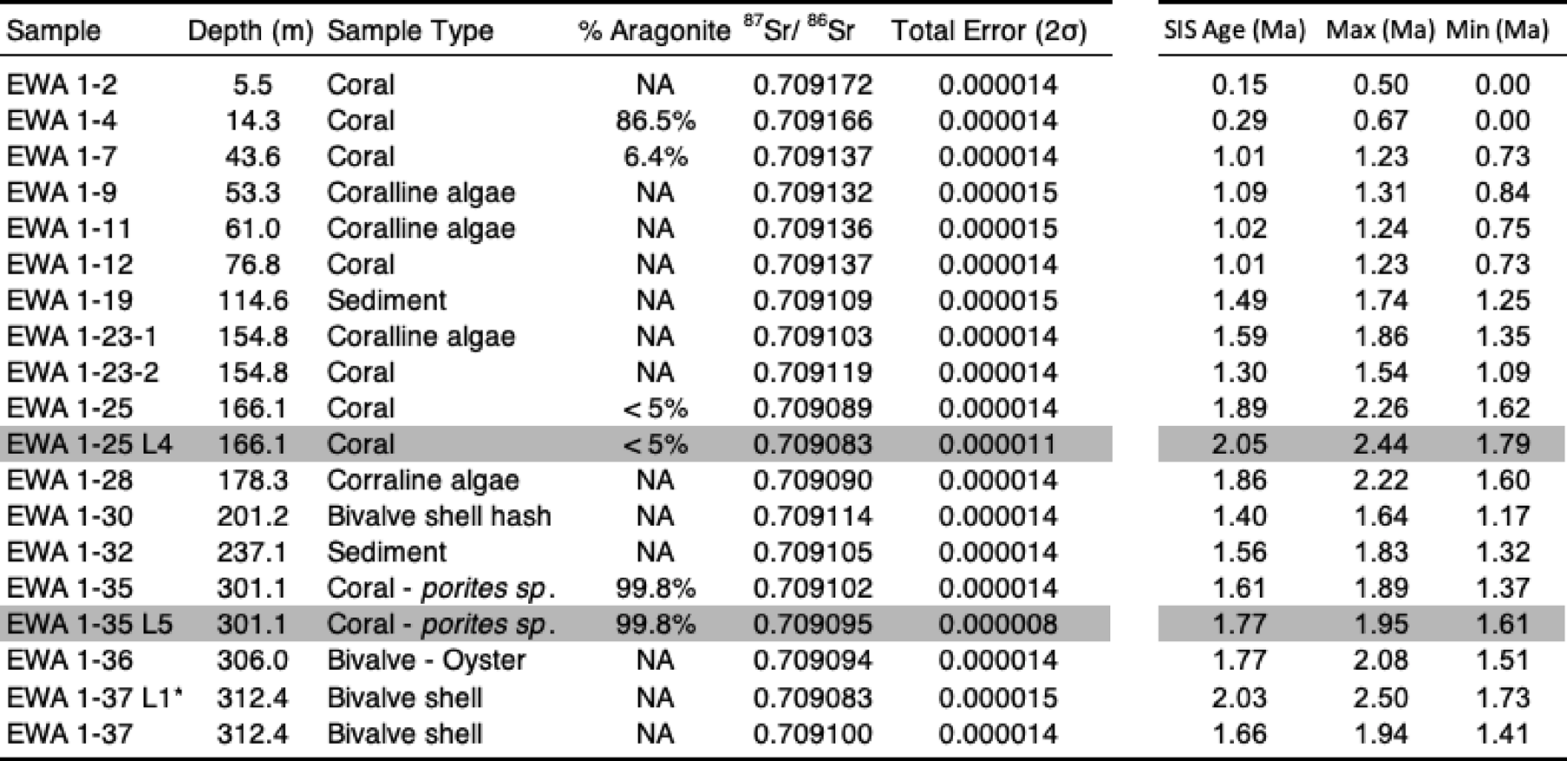
Strontium isotope stratigraphy from EWA-1.

Select corals analyzed with X-ray diffraction (XRD) for % aragonite vs. calcite at Lamont Doherty Earth Observatory, 2σ ranges of uncertainty (“errors”) =  ± 1%, NA = not analyzed. ^87^Sr/^86^Sr values given above were corrected for fractionation and adjusted for the NBS987 standard. Total uncertainty error reported above includes combined internal error and long-term NBS987 uncertainties. Shaded values measured at Stony Brook University (NBS987, all others were analyzed at the University of Texas at Austin). EWA1-37 L1* refers to the first wash (see text), thought to be more diagnostic of alteration fluid than the following split (EWA-1-37), and indicates that diagenesis might make samples appear older. An initial bulk rock sample at 247.5 m core depth (^87^Sr/^86^Sr = 0.709086 ± 0.000006), taken to assess the feasibility of this study, was not used in the above analysis.

**Fig. 4:**
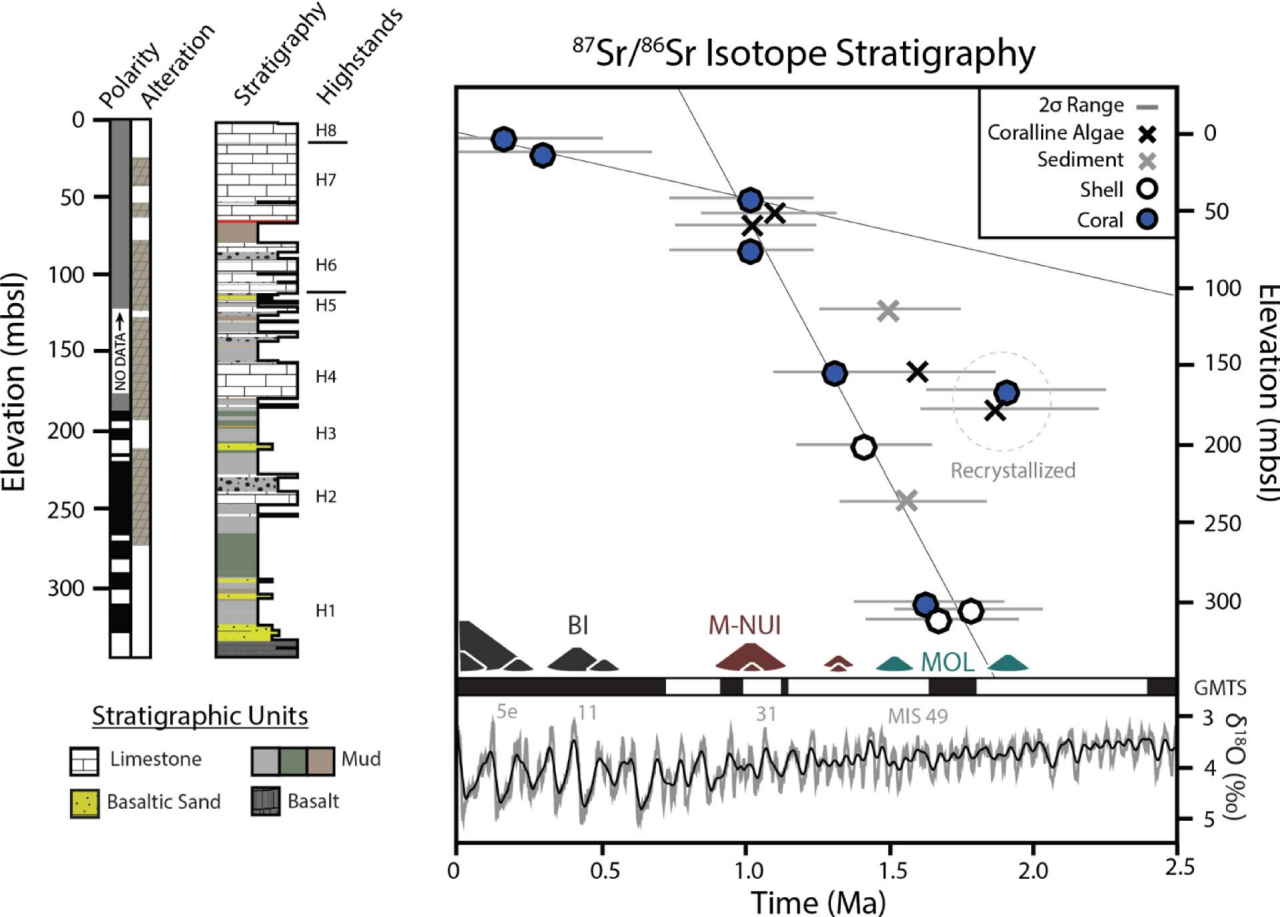
Stratigraphic description and ^87^Sr/^86^Sr chronology for EWA-1. (Left) Magneto-stratigraphy and alteration^25,32^. Stratigraphy based on descriptions of Stearns and Chamberlain^33^. Eight highstand sea level deposits identified by Resig^25^ are shown by ‘H’ symbol. (Right) ^87^Sr/^86^Sr isotope stratigraphy. Height of volcano symbols, including for the island of Hawai‘i (BI), Maui-Nui Complex (M-Nui), and Molokai (MOL), are proportional to the load of each Hawaiian Island^34^. (Bottom Right) Major geomagnetic reversals from Singer^35^ are given by black and white bars. The marine oxygen isotope stack of Lisiecki and Raymo^28^ is shown in gray and black (smoothed).

The resulting ^87^Sr/^86^Sr ages were compared to a range of possible vertical motion histories for the Ewa Coastal Plain simulated using the geophysical model developed for the Hawaiian Islands by Huppert et al.^13^. Each relative sea level (RSL) curve incorporates changes in eustatic sea level (Fig. 4, inferred from oxygen isotope records^28^) as well as vertical motion changes due to volcanic loading and plate migration over the Hawaiian Swell. The model was generated as follows: (1) a spatial median filter (480 km × 480 km) or spectral filter was used to separate Hawaiian bathymetry into its regional (swell) component and its residual component (the Hawaiian volcanoes and their compensation); (2) to estimate relative sea-level change due to flexural isostatic adjustment of O‘ahu to its own volcanic loading and loading of neighboring volcanoes, we assumed the lithosphere behaves as a continuous (unbroken) thin elastic plate, and we applied point loads corresponding to the weight of rock columns of height equal to the elevation of the residual bathymetry at each grid point on our digital elevation model. Note, however, these estimates to not account for viscoelastic relaxation of the lithosphere in our flexure modeling, which occur over million year timescales^29,30^. We used radiometric ages of shield-stage volcanic flows and mapped boundaries between each Hawaiian volcano to determine the time period over which the volcano (and corresponding point loads) was emplaced. We tracked the resulting displacement at the EWA-1 core site over time, assuming a constant loading rate during the shield building phase of each volcano. (3) ^87^Sr/^86^Sr ages were tested against models assuming effective elastic thicknesses of the lithosphere (*T*_*e*_) between 10 and 80 km. To estimate relative sea level change due to O‘ahu’s migration over the Hawaiian Swell, we tracked the elevation change between EWA-1’s present and past locations on the regional bathymetry, assuming a constant plate velocity^31^.

## Results

Strontium isotope data (Table 1, Fig. 4) may be used to divide EWA-1 into the following two chrono-stratigraphic units: (1) corals collected at 5.5 and 14.3 m core depth, within the uppermost carbonates , returned ^87^Sr/^86^Sr values ranging from 0.709172 to 0.709166, consistent with accumulation during recent interglacial highstands. (2) A lower chronostratigraphic unit below 43 m core depth contains carbonates with ^87^Sr/^86^Sr values ranging from 0.709137 to 0.709083, consistent with an early Pleistocene age.

The uppermost sediments (1–13 m core depth) of EWA-1 have been described as part of the Waimānalo formation by Stearns and Chamberlain^33^ which has been extensively dated to Marine Isotope Substage (MIS) 5 on the Ewa Coastal Plain and elsewhere^22,36^. Sample EWA 1–2 collected from 5.5 m core depth yielded a ^87^Sr/^86^Sr age of 0.2 Ma (2σ = 0–0.5 Ma). A thin band of fine-grained material at 13 m core depth, likely volcanic tuff, separates this material from potentially older (EWA- 1–4: 0.0–0.7 Ma) middle-Pleistocene material below. Veeh (personal communication^32^) reported a ^234^U/^238^U age of 140 ± 40 ka BP for an aragonitic sample collected at 17 m core depth in EWA-1 but anomalously high ^232^Th values prevented later attempts at U-series dating this unit (13–52 m core depth) by Easton and Ku^36^, who suggested an age > 300 thousand years (kyrs). Ten kilometers west of Ewa Beach, Sherman et al.^22^ identified a similar depositional sequence of limestone, dated to MIS 5, unconformably overlying middle-Pleistocene carbonates electron-spin resonance dated to 0.6 ± 0.1 Ma.

Below these uppermost carbonates, the Ewa Coastal Plain forms a wedge of early Pleistocene carbonates and clastic sediments that are up to ~ 300 m thick, overlying Ko‘olau basalt dated to 3.5–4.0 Ma^37^. Strontium isotope values from a well-preserved Porites coral (99.8% aragonite) and pristine oysters collected above (301–312 m core depth) the basaltic base of EWA-1 range from 0.709102 to 0.709094 suggesting an age of ~ 1.7 ± 0.3 Ma. Much of the basal section (~ 320–260 m core depth) also has normal magnetic polarity^32^, suggesting initial marine inundation of the EWA-1 core site during the Olduvai subchron (1.8–1.9 Ma). Accumulation of EWA-1 sediments after ~ 1.8 Ma is also consistent with an absence of diagnostic Pliocene nannofossils such as Discoasters^25^ and ^87^Sr/^86^Sr values (and inferred ages) of carbonates dredged from a drowned reef terrace 520–570 mbsl^10^ offshore our core site (14 km South), assuming superposition.

We suggest that diagenetic ^87^Sr/^86^Sr alteration, which most likely was derived from fluids flowing through the surrounding basaltic sediment (e.g., basalts of the Ko‘olau range ≈ 0.7040–0.7043^38^), lowered the strontium ratios of samples EWA-1- 25 and 28. An offset was found between the ^87^Sr/^86^Sr ratio of an oyster collected at 312 m core depth (0.709100) and its initial leachate (0.709083) value (Table 1), implying an age difference of roughly + 0.3 Myr, consistent with local diagenetic fluids incorporating the lower ^87^Sr/^86^Sr values associated with volcanic material. This data implies that alteration could cause sample strontium isotope ages to appear older, such as is observed in some of the more poorly preserved coral/coralline algae and sediment samples (Fig. 4; Table 1). However, similar to ^87^Sr/^86^Sr analyses of paired aragonite and calcite samples from Enewetak Atoll, Marshall Islands drill cores^39^, the strontium isotope composition of most samples collected between 43 and 300 m in EWA-1 appears to have been largely buffered by the surrounding carbonates and remain unaltered.

## Discussion

**Fig. 5:**
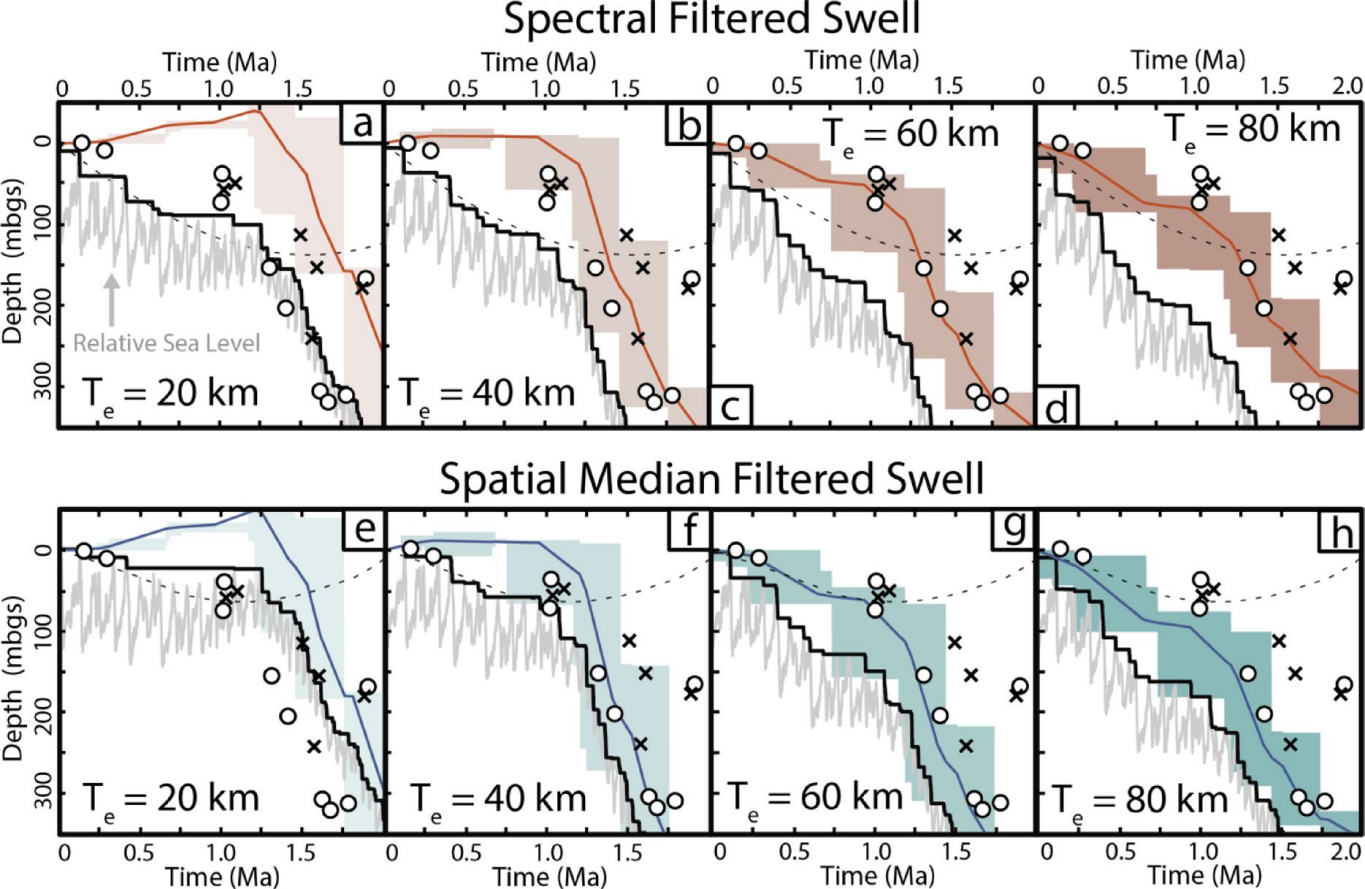
Comparison of ^87^Sr/^86^Sr dates to model generated RSL histories at the EWA-1 core site. RSL (jagged grey line) produced with T_e_ = 20, 40, 60 or 80 km (left to right). Black line tracks maximum (highstand) sea level through time. Deflection due to the site’s migration over swell topography with plate motion (grey dashed line) was computed using a spectral filter on upper panels (**a**, **b**, **c**, **d**) and a spatial median filter below (**e**, **f**, **g**, **h**) to isolate the Hawaiian Swell from the bathymetry. The time history of volcanic deflections at the core site is constrained by the blue or red boxed areas. Box height shows the total deflection due to loading of a volcano and box width shows the duration of that volcano’s shield building volcanism, with the trend assuming a constant deflection rate due to loading of each volcano shown by the overlain light blue or red lines. Circles and crosses use same ^87^Sr/^86^Sr symbols as in Fig. 4.

Figure 5 compares vertical motion-corrected highstand sea level through time simulated for effective elastic thicknesses *T*_*e*_ between 10 and 80 km to ^87^Sr/^86^Sr ages for EWA-1. The proposed accumulation history based on strontium isotope data is consistent with RSL histories modeled using elastic plate thicknesses between ~ 20 and 40 km and swell topography calculated using a spatial median filter (Fig. 5). RSL curves generated using T_e_ values greater than ~ 40 km fall well below the ^87^Sr/^86^Sr dated shallow water carbonates (e.g., sea-level limiting). Note that because strontium isotope analyses presented here were made on carbonates that accumulated in shallow water^25^, these carbonates must have formed at or below sea level and cannot be above our modelled RSL trajectories. Spectrally filtered swell topography also produces subsidence paths incompatible with minimum sea level datums recorded in EWA-1 from 1 Ma onwards. Previous work by Huppert et al.^13^ using this same model but independent sea-level index points (e.g. uplifted corals, drowned slope breaks) from across the Hawaiian Islands as a calibration dataset also constrained T_e_ to ~ 40 km. A similar value was found comparing an elastic plate model to seismic reflection profiles cross-cutting the Hawaiian ridge at O‘ahu^40^.

The simulated vertical motion of Ewa Beach, for viable elastic plate thicknesses and swell geometry, progresses from rapid subsidence to uplift as O‘ahu migrates away from the hotspot, consistent with available field data. Model and strontium-isotope data suggest subsidence of ~ 0.5 m/ka at Ewa beach between ~ 1.7 and 1.0 Ma largely due to the buildup of Lāna‘i (− 115 to − 99 m), West Maui (− 27 to − 8 m), Kaho‘olawe (− 73 to − 22 m) and Haleakalā (− 72 to + 29 m), assuming an effective elastic thickness between 20 and 40 km. Strontium isotope analyses of corals by Faichney et al.^10^ suggest that the site of drowned terrace formation in the Hawaiian Islands shifted from O‘ahu east to Moloka‘i in the early Pleistocene. Several of the drowned terraces found there may correspond with the eight transgressional cycles Resig^25^ identified in EWA-1 biostratigraphy. Likewise, slowing subsidence at Ewa Beach around 1 Ma likely represents a further shift of shield building to the east and the onset of drowned terrace formation near Lāna‘i^41^.

Over the last ~ 500 kyrs, subsidence during O‘ahu’s migration away from the crest of the Hawaiian Swell has been offset, in part, by uplift (+ 9–47 m for *T*_*e*_ = 20–40 km) from loading of the island of Hawai‘i, creating limited accommodation space for sediment accumulation at EWA-1. Raised shorelines on O‘ahu, also resulting from island uplift, have been extensively documented^42^ but caution should be taken in interpreting past eustacy from these features. Modelled vertical motion histories differ substantially across O‘ahu due to the position of relict shorelines on either the forebulge or within the flexural moat of the island of Hawai‘i volcanic loading during recent highstands.

## Summary

On the basis of existing literature and new data, the following simple depositional history is proposed for the Ewa Coastal Plain: (1) Prior to ~ 2 Ma, rapid subsidence due to crustal loading of nearby Moloka‘i produced a series of drowned offshore terraces^10^; (2) From 2 to 1 Ma, gradual westward migration of O‘ahu away from volcanism to the ‘southeast’ during construction of the Maui-Nui complex allowed shallow-water carbonate communities to become established and to keep-up with subsidence (~ 0.5 m/kyr); (3) After ~ 1 Ma, vertical motion created relatively little accumulation space for reef growth (< 50 m) as O‘ahu migrated off the apex of the Hawaiian Swell but was also uplifted by crustal flexure due to construction of the island of Hawai’i.

## Data Availability

All new data reported in this manuscript (e.g., 87Sr/86Sr measurements) are included in Table 1. Core material is archived at the University of Hawaii. For additional information, please contact mtoomey@usgs.gov.
